# Non-safety Assessments of Genome-Edited Organisms: Should They be Included in Regulation?

**DOI:** 10.1007/s11948-020-00222-4

**Published:** 2020-05-18

**Authors:** Bjørn Kåre Myskja, Anne Ingeborg Myhr

**Affiliations:** 1 Department of Philosophy and Religious Studies, Faculty of Humanities, NTNU, 7491 Trondheim, Norway; 2 GenØk-Centre for Biosafety, SIVA Innovation Centre, PB 6418, 9294 Tromsø, Norway

**Keywords:** Sustainability, Social utility, GMO, Ethical impacts, CRISPR, Norwegian Gene Technology Act

## Abstract

This article presents and evaluates arguments supporting that an approval procedure for genome-edited organisms for food or feed should include a broad assessment of societal, ethical and environmental concerns; so-called non-safety assessment. The core of analysis is the requirement of the Norwegian Gene Technology Act that the sustainability, ethical and societal impacts of a genetically modified organism should be assessed prior to regulatory approval of the novel products. The article gives an overview how this requirement has been implemented in the regulatory practice, demonstrating that such assessment is feasible and justified. Even in situations where genome-edited organisms are considered comparable to non-modified organisms in terms of risk, the technology may have—in addition to social benefits—negative impacts that warrant assessments of the kind required in the Act. The main reason is the disruptive character of the genome editing technologies due to their potential for novel, ground-breaking solutions in agriculture and aquaculture combined with the economic framework shaped by the patent system. Food is fundamental for a good life, biologically and culturally, which warrants stricter assessment procedures than what is required for other industries, at least in countries like Norway with a strong tradition for national control over agricultural markets and breeding programs.

## Introduction

Regulation of genetically modified organisms (GMOs) has mainly been concerned with safety for human health and the environment, expressed in strict assessment procedures. However, in some jurisdictions these procedures are supplemented with assessments of a broader nature, including societal issues. The paradigmatic example is Norway, where the Gene Technology Act (GTA) demands that sustainability, societal utility and ethical aspects are included in assessments of any GMO. However, recent technical developments within gene technology have challenged the regulatory frames for risk assessment, and may also pose problems for non-safety assessments. These new techniques have opened possibilities for editing genetic information and expression in organisms in a faster and more targeted way than previous methods. One of them is the genome editing technique clustered regularly interspaced short palindromic repeats (CRISPR), which has been rapidly adopted by academic and industrial research groups (Gratacap et al. 2019; Ledford 2017; Lusser et al. 2011; Paul and Qi 2016). Immediately one would think that these technologies are varieties of gene technology, and should be regulated accordingly. This is the conclusion of a recent ruling of the European Court of Justice (Court of Justice of the European Union 2018), stating that a genome-edited organism is to be regulated as a GMO. The decision was controversial, as genome editing can be used for mutagenesis purposes, where no novel DNA is inserted, resulting in an organism that may be identical with one that can occur spontaneously or that is produced by mutagenesis techniques (breeding techniques using chemicals that alter the structure of the DNA).

This article will discuss arguments concerning the inclusion of what Zetterberg and Björnberg (2017) have called ‘non-safety’ criteria in the regulation of genome-edited organisms, and explore under which circumstances assessment according to these criteria should be required in order to ensure that use of genome-edited organism are ethically justifiable and socially acceptable. Non-safety assessments are required for GMOs in some countries, such as Norway, where they are an addition to the compulsory risk assessment. When genome-edited organisms are regulated as GMOs, is the implication that they should also be subject to non-safety assessments as is required for GMOs? Underlying this legal question concerning inclusion in regulation there are ethical, societal, cultural and political arguments that will be explored in this article.

While Zetterberg and Björnberg (2017) focuses on the European Union (EU) regulatory framework for GMOs, this article will present the Norwegian Act and its system for assessment of sustainability, societal utility and ethics. This includes an overview of the current regulatory practice in Norway under the GTA, explicating normative bases for decisions in actual cases, and a discussion of whether it is possible to operationalize non-safety assessments, determining the feasibility of such assessments. Then it is discussed whether all genome-edited organisms, including those without added novel DNA, should be subject to non-safety assessments. This is the case if the ethical, social, cultural and political reasons for non-safety assessments regarding GMOs are valid also for all genome-edited organisms. Arguments for and against these requirements as they are expressed in the public and academic debate, internationally and in Norway, are analyzed in order to determine their relevance for regulation of genome-edited organisms. Although the GTA regulates all uses of gene technology, the majority of arguments supporting non-safety assessments are based on the use of these technologies in plants for food and feed production, and these applications will therefore be the focus of this article. The discussion aims to demonstrate that it is feasible and justified to subject GMOs to the non-safety assessments required by the GTA, and that arguments for such assessments are valid also for genome-edited organisms. The central arguments for the inclusion of genome-edited organisms in non-safety assessments in the Norwegian context are the disruptive potential of genome editing in food and feed production, as well as the socio-economic framing of these technologies. They are patent-protected technologies within an area considered to be of crucial significance for society. Food production is essential for human survival, which warrants stricter public control than what is required for other industries. Given the generally supported Norwegian model for protection of food production, demanding non-safety assessments is warranted.

Although the argument states that non-safety assessments are feasible and justified within the Norwegian context, a different matter is whether such assessments should be implemented in regulation of GMOs and genome-edited organisms everywhere. As the arguments below are partly based on the particular cultural and ethical traditions of Norway, they do not provide sufficient basis for a general world-wide adoption of such assessments. However, several countries do have analogous traditions that support implementation of non-safety assessments. In addition, several of the arguments are of general validity and may justify that the issue is considered also within jurisdictions that have no similar common tradition for protection of food production.

## The New Genome-Editing Techniques for Crop Breeding

There is a continuous drive to improve crops by breeding. Genetic modification enabled a more powerful and directed method for introducing novel traits in plants, and at present the most commercially successful GM crops are herbicide tolerant, insect resistant or a combination of these traits (ISAAA 2018). Recently, new plant breeding techniques (NPBTs) have been developed (Globus and Qimrom 2018; Lusser et al. 2011). All of these can introduce breaks in the DNA at target sites followed by stimulation of the organism’s DNA repair mechanisms. New genes from the same or other species can then be inserted, or parts of the organism’s DNA can be deleted. If new genes are not inserted, the DNA repair mechanisms may lead to a mutation causing reduced function or no expression of a specific gene. Among the NPBTs, the CRISPR system has achieved highest interest as a genome-editing tool since it is easy to design, produce and use. The CRISPR system is at present widely used at universities, research institutes as well as industrial research groups.

Arguably, most public interest and debate has been generated by the medical potentials of genome editing, but there is well-founded hope for important contributions within agriculture, aquaculture and food production (Barrangou and Doudna 2016; Belhaj et al. 2013). Their use may enable humanity to meet the challenges of climate change and increasing populations by developing high-yielding, saline- and drought-tolerant or virus- and disease-resistant plants (Khatodia et al. 2017; Zaidi et al. 2016). Furthermore, its use may improve efficiency in animal husbandry by developing parasite- and disease-resistant animals, as well as hornless cows and sterile fish (Gratacap et al. 2019). The techniques can be used to combat diseases or pests with gene drives, and do also hold promises for animal breeding to improve the safety for growth of organs to be used in humans for xenotransplantation purposes, to mention some of the current research interests.

## Regulations of GMOs and Genome-Edited Organisms

The pressing question currently is whether genome editing techniques create organisms that fall under or should be exempted from current GMO regulation (Ishii and Araki 2017; Nature Editorial 2015). Related questions concern whether current regulatory frameworks must be revised and adapted to these new techniques to ensure adequate handling of the techniques themselves, as well as the resulting products. Arguments for exempting genome-edited organisms from regulation include: (a) the analogy to organisms found in nature, (b) the similarity to organisms originating from mutagenesis techniques, and (c) the excessive regulatory burden placed on GMOs that is stifling innovation as well as global trade of agriculture commodities (Jones 2015). The argument with most impact is the second, stating that the products from genome-edited organisms are indistinguishable from products created by processes already excluded from legislations, as for example in the EU Directive which excludes mutagenesis. It is therefore argued that the harm potential of genome-edited organisms is equal to that of these non-regulated organisms (Globus and Qimrom 2018; Hartung and Schiemann 2014).

The regulatory systems for GMOs differ across the world (Ishii and Araki 2017). They are commonly classified as either process- or product-based. Many countries have implemented a GMO regulatory approval before environmental release and use of GMOs, where the main element is an assessment of human safety and environmental risk. Since these countries make the technology basis for regulation, they are commonly considered to be process-based. A typical example is countries within the EU, as well as Norway, where GMOs are subject to an extensive risk assessment procedure on a case-by-case approach, conducted by the European Food Safety Authority (EFSA). As mentioned above, this regulation will include genome-edited organisms, but this conclusion was not obvious. While waiting for the decision by the European Court of Justice, some countries used their national legislation as basis for regulation. For example, Sweden decided that genome-edited organisms with no inserted foreign DNA could on a case-by-case decision be exempted from GMO regulation, while those that have novel genetic material inserted, will be regulated and labelled as GMOs. However, the European Court of Justice ruling (Court of Justice of the European Union 2018) made it clear that the established EU exemption of mutagenesis is only relevant for organisms obtained through methods of mutagenesis that have been conventionally used in a number of applications, giving them a history of safe use. NPBTs are not covered under this exemption. This implies that all applications of NPBTs will follow a case-by-case procedure and be risk assessed according to guidelines under the GMO Directive (EU-Directive 2001).

Other countries that have a variety of process-based regulation, such as the USA, have decided to regulate GMOs under existing frameworks, applying the principle of substantial equivalence, i.e. taking conventional varieties as the basis for risk assessment of novel products or traits (Ishii and Araki 2017). Genome-edited plants are not subject to specific regulatory requirements as long as they have no novel traits due to genetic modification, such as expressing herbicide tolerance or antibiotic resistance. In 2010 the Animal and Plant Health Inspection Service under the US Department of Agriculture made the decision to exempt herbicide-tolerant maize generated by NPBTs from regulation. The first CRISPR genome-edited plant exempted from regulation was a mushroom variety with prolonged shelf life due to reduced browning process. Later CRISPR-edited maize with changed production process of starch, developed by Dupont, has also been exempted from regulation (Waltz 2016). Genome-edited animals, however, are included in the Food and Drug Adminstration’s safety assessment procedure, which enabled them to disclose unintended alterations in genome-edited hornless bulls (Hahn 2020).

In product-based systems, where Canada is the paradigmatic case, any “plant with a novel trait” is subject to risk assessment. The trait must be new to the environment and must also be considered to impact on how the plant is used, as well as on its health or environmental safety. Such a plant may be a GMO, a plant with induced and normal mutations, or even a plant produced by traditional breeding (Smyth 2017). This will of course mean that there will be no special regulation for genome editing. Argentina, which has a process-based system, was the first country to adopt a regulation of NPBTs (Resolution no. 173/2015), deciding that some of the genome-edited products that do not contain a transgene are exempted from GMO regulation (Whelan and Lema 2015).

The same regulatory patterns are followed concerning labelling requirements. In all countries within the EU and in Norway, any approved GM plant or food product must be labelled if it contains more than 0.9% of a GM ingredient, and this will be required also for genome-edited products. The USA is in the process of implementing a national “GE disclosure standard” (Bovay and Alston 2018), while Canada have no required labelling, merely regulations for voluntary labelling of food as GM or non-GM. In these jurisdictions, genome-edited organisms and products thereof that are exempted from regulation will not be labelled.

As stated above, EU and Norwegian regulation is usually called process-based, justified in the precautionary principle, while Canada’s approach is said to be product-based, using substantial equivalence as the main point of reference, and the regulatory principle of the US is described as a combined approach (Ishii and Araki 2017; Zetterberg and Björnberg 2017). It is, however, more correct to say that the EU and Norway use process as a criterion for safety assessment, whereas the assessment itself is product-based. Thus, they also have a combined process- and product-based approach.

The regulatory approach is significant for non-safety assessments: In a purely product-based approach, either all or no novel organisms should be subject to non-safety assessments, regardless of breeding approach. In a process-based or combined regulatory system, one can argue for non-safety assessment of products based in certain technologies without requiring the same assessments for products developed by conventional methods. This article will defend a distinction of this kind.

## The Inclusion of Non-safety Assessment in GMO Regulatory Frameworks

Some countries, such as the USA, have regulations that primarily consider safety and efficacy, while other jurisdictions include non-safety factors. The relevance of non-safety assessment has been recognized in international frameworks (e.g. article 26 of the Cartagena Protocol on Biosafety), and in European and African fora (Binimelis and Myhr 2016; Spök 2010). In Europe, a new directive on GMOs was approved in 2015 (Directive (EU) 2015/412), amending Directive 2001/18/EC to allow a Member State (or region) to adopt measures restricting or prohibiting the cultivation in all or part of its territory of a GMO, or of a group of GMOs defined by crop or trait, based on grounds such as those related to socio-economic impacts, avoidance of GMO presence in other products, agricultural policy objectives or public policy (Directive 2001/18/EC Article 26b). However, this is optional, so there is no requirement to perform an assessment of socio-economic impacts or to take such assessments into account during the approval process in EU. Although several countries include non-safety assessments in their GMO evaluation, Norway is arguably the country with most experience with such evaluations (Marcoux et al. 2013; Roger 2015). The Norwegian Act of 1993 requires consideration of ethical issues, societal utility and contribution to sustainability of GMOs, as well as of direct and indirect impacts on agricultural practice of GMOs in the assessment processes.

The inclusion of non-safety considerations is highly controversial (Zetterberg and Björnberg 2017), and there is no consensus on the topics that should be included in such assessments. Issues of debate include scope, methods and disciplines involved, timing of consideration, baselines and comparators, indicators, the relationship with other fields of knowledge and with other dimensions of risk assessment (Binimelis and Myhr 2016; Falck-Zepeda and Zambrano 2011; Greiter et al. 2011; Spök 2010). These are challenges that need further elaboration with the aim to establish methodologies to be used during the processes of framing, data gathering, assessment and decision-making (Catacora-Vargas et al. 2018). The current Norwegian approach to non-safety assessments provides examples of how this could be done, and serves as an appropriate background for an analysis of the question whether also genome-edited organisms should be subjected to this kind of assessment when they are required for GMOs.

## The Norwegian Gene Technology Act (GTA): Sustainability, Societal Benefit and Ethics

The GTA of 1993 regulates the production and use of GMOs. The first paragraph states: “The purpose of this Act is to ensure that the production and use of genetically modified organisms and the production of cloned animals take place in an ethically justifiable and socially acceptable manner, in accordance with the principle of sustainable development and without adverse effects on health and the environment” (GTA 1993, §1). More specifically, the Act lays down that GMOs may only be approved when there is no risk of adverse effects on human or animal health or the environment, and that “considerable weight shall be given to whether the deliberate release will be of benefit to society and is likely to promote sustainable development” (GTA 1993, §10.2).

The Norwegian Biotechnology Advisory Board (NBAB) is responsible for making a broad assessment of GMOs, and has a special responsibility for assessing sustainability, social benefit and ethics. It was early established that the societal utility criterion should only be relevant for impacts of the product within Norway, whereas sustainability and ethics may take into consideration factors that may evolve over longer term, with an impact on global developments (NBAB 2018a). In 2000, the Board published a report on how to operationalize the concepts of sustainable development, social benefit and ethical and social considerations in the GTA (NBAB 2000). Parts of this report were included in the appendix of the Regulations on Impact Assessment pursuant to the GTA in 2005. Since 2000 there has been a broader scope and more depth of detail in the assessment of GMOs by the Board (Roger 2015; Rosendal 2008), moving from only considering societal concerns related to pesticide use to include benefits to the community, opportunities to reuse seed for farmers, ethics and sustainable development. This is also reflected in revisions of the report carried out in 2006 and 2009.

In 2009 the Norwegian Environmental Agency requested a report on how and to what extent information in marketing applications for GMOs fulfill the criteria of sustainable development and societal utility in the GTA. In this report, two GM plant marketing applications were analyzed and it was found that the information supplied by the applicants can be used for answering issues of relevance for environmental and health risk assessment, while little of the provided information can be used for assessing how the GM crop might contribute to sustainability and societal utility (Rosendal and Myhr 2009). This report was followed by a request from the Norwegian Environment Agency to the NBAB to carry out a project aimed at operationalizing the concepts of sustainable development. The outcome was two reports: one on sustainability connected to insect-resistant GM plants (NBAB 2011), and one on sustainability by herbicide-resistant plants (NBAB 2014). The Board invited representatives from different institutions to contribute as ad-hoc experts, and the parameters they elaborated included environmental, societal and economic aspects (see Table 1).

**Table 1:** Parameters and example of questions in the guidelines elaborated by the NBAB for conducting assessments of contribution to sustainability. Adapted from the NBAB (2011, 2014)

Parameter	Questions
Environment/ecology	On the GM plant: -Gene flow to wild and agricultural relatives -Interaction between plant and the environment -Impacts on preservation of biodiversity -Available comparison with control plants On the herbicide/Bt toxin: -Effects of altered use by the GM plant -Development of resistance in wild and agricultural relatives Impacts on soil, water, energy and climate in area of GM crop cultivation
Society/economy	Access to sufficient, safe and healthy food (food safety, food security and food quality) at the local and regional level Animal health and welfare by consumption of GM plant-based feed Living conditions and profitability for the farmers who cultivate GM plants, in the short term (less than 5 years) and in the long term (more than 20 years) Health and safety of farmers cultivating GM plants Contracts and framework conditions for farmers cultivating GM plants Increase or decrease of employment opportunities in the area Use of agronomic factors, and developments of costs and incomes at the farm level by GM plant cultivation Access by farmers to seed and ownership issues

In 2018, on request from the Norwegian Environment Agency, the Board issued a third report on how to use the societal utility criterion in assessments of GMOs (NBAB 2018b). The recommended procedure is based on principles for socio-economic cost–benefit analyses, although the difficulty in ascribing economic value to some crucial societal benefits is recognized. In the end, decisions in hard cases will still be a matter of prudential deliberation rather than economic calculation. Hard cases are those where it is unclear whether the criteria are fulfilled in a sufficient manner, as well as those where there is a positive conclusion on one criterion, but negative on others. A product can for example be socially beneficial, while having negative impact on sustainability. Finally, a report on operationalization of the ethics criterion was worked out by a group of bioethics scholars on commission by the Norwegian Environment Agency in 2019. This is currently under evaluation by relevant authorities.

Many involved parties in Norway have interpreted the Act to state that it is not sufficient that the non-safety contribution of the new product is as good as existing alternatives. The product must be considered an improvement when compared to existing varieties. However, the Ministry of Climate and Environment has made it clear that this is not the official understanding. The Act literally states that “considerable weight” (GTA 1993, §10.2) shall be given to the non-safety issues, leaving it open how this should be interpreted. One could, on the one hand, argue that there is no reason for introducing competing products that are no better than the existing, as long as there is wide public resistance to the technology in question. On the other hand, one could say that this is contrary to generally accepted principles of free competition. As long as products do not harm health or environment, or there is no reasonable concern about long-term negative societal impact, one should let the market decide; an argument supporting a neutral interpretation of the requirement.

When regarding concrete recommendations issued by the Board on marketing applications based on the non-safety criteria, a few important points are worth noticing. First of all, until now there have been no GMO applications directed specifically to Norway; applications assessed by Norwegian authorities are those that have been submitted to the EU. All GMOs approved in the EU are approved in Norway, unless there are specific reasons based in the GTA criteria for rejecting them. In the following, the discussion will only concern those applications that have been approved by Norway, which are five carnations, and those cases where Norwegian regulation deviates from the EU decisions, in total 10 GM plant applications. Before an analysis of the non-safety assessments is presented, the procedure and problems in acquiring relevant information concerning the non-safety criteria will be described.

### Difficulties in Acquiring Relevant Non-safety Data

The working procedure by the NBAB, when receiving a new application for use and release of a GMO, starts with drawing up a list of concrete questions regarding the sustainability and societal impact in light of the introduced traits. This list is based on the Board’s reports on sustainability (NBAB 2011, 2014) and on the societal criterion (NBAB 2018b). The list has also been submitted to some of the applicants by the Ministry of Climate and Environment or the Norwegian Environment Agency (Marcoux et al. 2013; NBAB 2017a, 2018c), but has, however, in most cases not been answered. One can regard this as surprising, considering that this is a chance for the applicant, which in most cases is a biotech company, of proving that the specific GMO actually provides significant and valuable improvements for the environment and the society at large. However, there are also possible reasons for not answering the questions, even if it is assumed that the outcome of the inquiry into the performance of the variety would be positive. First, Norway is a small and insignificant market, and there are few incentives for spending resources on answering questions only relevant for approval and entry in this country, with a possible exception for products that target specific niche markets. Second, answering questions could be taken as an acceptance of the relevance of non-safety assessments for approval of GMOs, with consequences for other varieties and for other, more important markets. It could be interpreted as an implicit acceptance that GMOs are different from other commercial products in terms of sustainability, societal benefits and ethics. If the applicant did answer the list of questions, they would have to provide a justification if they thought some questions were irrelevant or based on what they held to be unacceptable premises, and that would be taken as an implicit acceptance of the legitimacy of asking these questions, and hence an encouragement for inclusion of such non-safety factors in regulation.

One could argue that the producers’ reasons for complying with safety assessment requirements are relevant for complying with non-safety assessments, as well. All food is subject to safety measures. A particularly thorough assessment of GMOs can be in the interest of the applicant for two reasons: (a) it can be a marketing argument for choosing this product, and (b) it can be a way to avoid liability in case some harm should be documented afterwards. Certainly, the first argument could be of theoretical relevance for non-safety assessments, but probably with less effect in practice. Is it likely that a significant group of people will prefer a GM maize variety because it has been approved as more sustainable than the non-GM alternative? The second argument is not relevant—it is wholly unlikely that a biotech company would be sued for selling a non-sustainable GM variety. In short, the applicants do not have strong incentives for contributing to non-safety assessments.

It is conceivable that a producer did embrace non-safety assessments for moral reasons, for example in order to take responsibility as part of a corporate strategy, or because they consider that they ought to take responsibility for a sustainable future in line with one way of interpreting Jonas’ (1979) notion of a forward-oriented imperative. However, current politics of the dominant biotech corporations give little indication of such concerns, although some have adopted corporate social responsibility strategies highlighting sustainability. One may assume that they prefer to define their own concept of sustainability and ethics, rather than subjecting themselves to an external assessment, for reasons mentioned above.

The lack of cooperation from the applicants means that the Board must make non-safety assessments without relevant information from the applicant beyond data already available in the application, which is challenging. They lack resources to conduct empirical studies of the actual use and effects of the product or similar products. Much of the effects on sustainability are of a long-term nature, and even if the GM plant has been cultivated and used some years in other places of the world prior to the final approval process in Norway, the evaluation of sustainability will be mainly of a prognostic nature with significant uncertainty. It will be based on information on long-term effects from different climatic and growth conditions if such is available, and on theoretical studies. Still, the operationalization documents (NBAB 2011, 2014, 2018b) provide guidance and can arguably form the basis for equal treatment of comparable products and predictability, as is indicated in the following descriptions of cases where Norway has made a decision.

### Approval of Five Carnations: Non-food GMOs

Thus far, all GM food applications to Norway have been rejected based on risk assessments or issues of precaution, ethics, sustainability or societal effects. The only non-prohibited GM plants under the GTA in Norway are five carnations modified to express a new colour, such as pink, blue or purple.

It is interesting to note that the only applicant that has answered the NBAB sustainability and benefit questions is the producer of some of these carnations (NBAB 2017a, 2018c). In this response, the producer gave a textual account of how they have implemented measures to increase the product’s sustainability. The producer argued that the plant has increased societal benefit by making new colours of the flower available in the market, and that the product increases knowledge in biology, biosafety and related areas. In addition, increased local employment and better wages for the workers were mentioned. This demonstrates that it is possible to give an argumentative account of the positive non-safety effects of a GM plant that could be considered in the Norwegian approval process. However, the arguments were not considered decisive for the positive recommendation by the NBAB, since the producer’s sustainability arguments could not be substantiated with data from a comparison with a non-GM carnation. The Board considered therefore that the argument could be used for any carnation production and were not specifically related to the GM plant. Furthermore, any new successful production would improve employment, and the availability of new colours in an already varied flower market was considered a negligible benefit. Thus, the arguments provided by the carnation producer were, according to the NBAB, of little relevance for approval according to the Act.

One can criticize the Board for putting too little weight on arguments concerning employment and a varied market, as this successful production was a direct result of genetic modification. The important point in this context, however, is that the response demonstrates that it is possible for applicants to handle non-safety related questions in a way that can be illuminating for the approval procedure. For a GM plant with more impact on sustainability and societal issues one could build a stronger case for positive non-safety impact.

It is also interesting to notice that in approving these carnations, the Board’s majority abandoned its practice of demanding a positive contribution to sustainability or society, arguing that these plants are of little significance and it therefore seemed excessive to prohibit them (NBAB 2016, 2017a, 2018c). This has been criticized for being inconsistent, and some of the members of the Board have expressed that they should have voted against the approval, confirming that they agreed with the critique. However, one could also interpret the decision as introducing an implicit, pragmatic principle that non-safety issues should be applied leniently for products not used in food or feed that have low impact on environment and society. This is in keeping with the idea that GM products should be treated on a case-by-case basis, and may create precedence for the non-safety assessment of future genome-edited organisms with low risk.

### Spread of Resistance Genes into the Environment: Safety and Non-safety Concerns

The main argument for prohibiting some GM plants in Norway has been a concern with environmental spread of antibiotic resistance genes. Norway has a very strict policy on the use of antibiotics and has established strategies to avoid an increase in antimicrobial resistance, which is the reason for prohibiting GM chicory, one GM maize and three GM oilseed rape plants (see Table 2).

**Table 2:** GM plants prohibited in Norway (based on Lovdata)

GMO case (event/name)	Prohibited in Norway	Reason for prohibition in Norway
GM chicory (RM3-3, 3-4, 3-6)	01.10.1997	Risk to health for humans and animals. Contains antibiotic resistance genes that can spread to pathogenic bacteria. No social utility and access to alternative productions systems
GM maize (Bt176)	01.10.1997	Risk to health for humans and animals. Contains antibiotic resistance genes that can spread to pathogenic bacteria. No social utility and access to alternative productions systems
GM oilseed rape (MS1xRF1(PGS1))	01.10.1997	Risk to health for humans and animals. Contains antibiotic resistance genes that can spread to pathogenic bacteria. No social utility and access to alternative productions systems
GM oilseed rape (MS1xRF2(PGS2))	01.10.1997	Risk to health for humans and animals. Contains antibiotic resistance genes that can spread to pathogenic bacteria. No social utility and access to alternative productions systems
GM oilseed rape (Topas 19/2)	14.12.2012	Risk to health for humans and animals. Contains antibiotic resistance genes that can spread to pathogenic bacteria. No social utility and access to alternative productions systems
GM oilseed rape (GT73)	14.12.2012	Risk to environment by spread of the GM plant or by the transgene. No social utility and access to alternative productions systems
GM maize (1507)	02.06.2017	Ethical reason. The GM plant is tolerant to the herbicide glufosinate ammonium. This herbicide is prohibited in Norway. No social utility and access to alternative productions systems
GM oilseed rape (Ms8)	02.06.2017	Risk to environment by spread of the GM plant or by the transgene. No social utility and access to alternative productions systems
GM oilseed rape (Rf3)	02.06.2017	Risk to environment by spread of the GM plant or by the transgene. No social utility and access to alternative productions systems
GM oilseed rape (Ms8xRf3)	02.06.2017	Risk to environment by spread of the GM plant or by the transgene. No social utility and access to alternative productions systems

In a health perspective, antibiotic resistance genes in food and feed production should be avoided since a potential spread of resistance can represent a problem for medical and veterinary treatments and have social costs. There is an increasing awareness that antimicrobial resistance is not only a health issue but also an environmental problem. In Norway’s strategy against antimicrobial resistance it is emphasized that safe food shall be produced in a sustainable way, and the prohibition of GM varieties containing antibiotic resistance marker genes is thus partly based on the sustainability criterion in the Act. Although this is treated as a matter of risk, and hence, falls within the safety issues, the possibility for persistence in the environment, uptake in pathogenic bacteria and the subsequent implications is not quantifiable without significant information currently not available, and is hence a matter of societal impact and sustainability.

As is pointed out by several authors, including Brian Wynne (1992) and Sterling (2007), the scientific concept of quantifiable risk is hardly adequate in order to handle a situation of potential harm in areas with scientific uncertainties. This blurry line between risk and sustainability demonstrates the significance of including non-safety issues in order to make a decision that is socially acceptable. This overlap between a standard risk assessment, areas of uncertainty and ignorance (Wynne 1992; Stirling 2007; Stirling et al. 2018) is highly relevant for the issue of spread of transgenes, such as antibiotic resistance marker genes, as well as herbicide tolerant genes.

The environmental risk of spread of herbicide tolerant genes to weedy relatives and non-GM plants was important for prohibiting four GM oilseed rape varieties (see Table 2), as oilseed rape has wild relatives in Norway. Herbicide tolerance in weeds has been reported, with an increased total use of herbicides following the introduction of GM varieties. The uncertainties concerning long-time effects on herbicide use for herbicide tolerant varieties were the decisive arguments for the NBABs holding that plants with these characteristics do not overall contribute to improving sustainability (NBAB 2017c, d). The Board did consider that reduced tillage could entail positive sustainability effects, but since no documents supporting this were provided by the producers, the negative sustainability effects were considered decisive.

Norwegian GM regulation is committed to the Precautionary Principle, according to the Preamble to the Act, which means that reasonable doubt as to the sustainability of the product provides a basis for prohibiting it, as long as no other significant consideration affects the evaluation. Hence, plausible accounts of uncertainty and possible ignorance concerning adverse environmental effects will be decisive in the absence of evident benefits. However, one factor that could have altered this negative assessment, is the societal impact of GM plants for salmon feed in Norway.

### Conflicting Societal Goals in Assessments of Non-safety Factors

There is a potential conflict in Norway between rising costs of non-GM fish feed and the salmon farming industry assumption that public resistance to GM would have a negative impact on sales of GM fed salmon.

At present almost 80% of the soy cultivated is GM (ISAAA 2018), which makes it increasingly difficult to ensure sufficient supply of GM free feed in Norwegian aquaculture. In addition, according to the Norwegian feed importer Denofa, measures to ensure that the imported soy is GMO free, such as testing and co-existence measures, increase the price difference and makes the price of non-GM soy 7–10% higher than of GM soy (B. R. Thomsen, personal communication). This is the reason why four fish feed producers had a standing exemption from Norwegian GM regulation for 6 years concerning processed material from 19 different GM plants based on a concern for potential shortage in available non-GM feed on the market. In 2014, the exemption was discontinued as it was never used (Norwegian Food Safety Authority 2014). The producers’ stated reason for not using this possibility was fear that use of GM-based feed would have a negative impact on the sale of Norwegian salmon.

There is still no pressing demand for GM soy, so there is no recognizable societal benefit from allowing import. Denofa’s contracts with soybean farmers in Brazil secure the import of GM free soy. At present this is manageable, but further increased price difference may make the Norwegian salmon industry less competitive unless they start using GM soy or find alternative feed sources. Thus, the Norwegian salmon industry may in the future need to explore whether their assumption about public resistance to GM-based feed in aquaculture is correct, and to calculate the costs and benefits of leaving their non-GM strategy. If they find that benefits outweigh costs, they may wish to start using GM-based feed in part of their production. This could become a decisive positive element in the assessment of the societal benefit of GM plants. It would, however, still be open how the balancing of sustainability and societal benefit would play out.

### The Ethics-Based Rejection of GM Maize

Recently, the Norwegian government decided to prohibit the import of one GM maize variety, 1507 (see Table 2). This variety is approved for use in food and feed, but not allowed for cultivation in Europe. It is resistant to glufosinate-ammonium, a herbicide that is not allowed for use in Norwegian farming because it is considered to be toxic to humans and other mammals. The Board recommended that this variety and other varieties with the same trait acquired by genetic modification should not be approved. They argued that it is ethically unacceptable to import products created for use with an herbicide that is prohibited in Norway (NBAB 2017b), even if the farming is done in countries where glufosinate-ammonium is allowed. This would amount to accepting others to take risks that are considered unacceptable in Norway, and hence an expression of double standards. It was also emphasized that there is at present no societal benefit in the use of GM maize within Norway.

This advice by the Board may appear to be inconsistent since Norway imports many non-GM agricultural products that have been subjected to herbicides and pesticides not presently approved for use in Norwegian agriculture. The morally relevant difference, however, appears to be that the specific *intention* of these GM varieties is that they are to be cultivated with the use of glufosinate-ammonium. That is not the case with the imported non-GM varieties. The Board’s recommendation based on the ethics criterion was followed by the government in their prohibition of the GM maize (Ministry of Climate and Environment 2017), and was extended to other varieties and plants with resistance to the same herbicide. The Norwegian Environment Agency has recently recommended that also the cultivation of this GM maize and another GM maize (Bt 11) modified to tolerate glufosinate-ammonium, should be prohibited (Norwegian Environmental Agency 2019).

The cases described above show that all three non-safety criteria in the GTA, sustainability, social benefit and ethics, have been used in the decisions where Norway has approved or prohibited GM products. The advice of the NBAB and the decisions made by the competent authorities also illustrate how the criteria have been used, and indicate what is required for assessments of GM applications. Implicitly in these decisions there are several cultural presuppositions and normative arguments that are further analyzed below.

## The Cultural Basis for Non-safety Assessments

The regulatory system for GMOs should not be regarded in isolation from the economic and sociocultural significance of food production, a point recognized in the amended EU Directive 2001/18/EC. Food is not like any other product, since it is the fundamental factor for human survival. Therefore, there are reasons for better public control over food production than what is required for other industries. This public control has found its particular expression in the social democratic corporative nature of the Norwegian food production system, where the state, farmer’s organisations and the major food producers cooperate closely (Terragni 2006). The aim has been to ensure food safety, food security and affordable food nationwide, as well as ensuring that farmers have a market for their products. Although this virtual monopoly has been loosened the last decade, allowing some competition from private companies, it is still the dominant characteristic of Norwegian food production. This corporative character of food production extends to the breeder’s organizations, such as Geno, Norsvin and Graminor, where there has been close cooperation between farmers’ organizations and public research institutions, ensuring high quality and robust animals and plants that have been attractive for breeding internationally.

Although food safety and environmental risk are primary concerns, there are other legitimate interests for consumers and society when considering the acceptability of novel foods. Food has cultural significance, being part of human self-understanding and belonging to local traditions and ways of life. A number of consumers choose foods based on social, political and cultural reasons, as is seen from the relative success of voluntary labelling initiatives for fair trade and organic products. The cultural meaning of food and food production is exemplified in organic farming, “Local Food” (Martinez et al. 2010) and “Slow Food” movements (Gaytán 2004), as well as in the protection of regional characteristics of agricultural products through standards such as the EU quality schemes, the Norwegian “Spesialitet”, and voluntary standards. These movements and classification systems all combine a series of conceptions of the quality and safety of food being dependent on a certain relationship between the farmer, the land and local traditions that are perceived as tokens of quality and safety of food, what could be termed ‘specialty food’ (Halkier et al. 2017). Thus, the issue of trust is crucial for the impact of these still fairly marginal, but influential approaches to agriculture (Myskja 2015), not least in Norway, where ideas of local food and traceable origins has had especially strong traction (Halkier et al. 2017).

Both proponents and adversaries of gene technologies in agriculture present this technology in a radically different way compared to the conceptions of food presented above. Agricultural biotechnology has until now been a technological solution for large-scale production of food in high-input, industrialized agriculture. In 2018 50% of all commercialized GM crops were soybean (ISAAA 2018), a plant used in feed and processed food. Maize is the second most common. The major trait is herbicide tolerance and second comes stacked traits, which is a combination of herbicide tolerance and insect resistance, hence most GM crops are developed to suit this type of agriculture. As the proponents will point out, most of the food and feed produced in the world today is produced in this way, and their claim is that this is the only viable approach to sufficient food production for a growing world population. It is typically the product of monocultures in high-input industrialized agriculture, where the plant varieties are developed through a number of modern breeding techniques, including hybridization and mutagenesis. The GM technology is on this conception, a clear contrast to farming as a “focal practice” in the sense of constituting a meaningful practice rather than serving as a mere means for producing food (Thompson 2000). One could claim that the current GM crops present a paradigm case of decontextualized food production, where elements associated with culture-based trust in the quality, safety and sustainability of food do not play important roles and where there is little interest in assessing ethical and social impacts. Now, GM crops are increasing their share of the total food production, and industrialized agriculture is not synonymous with GM production, just as GM production is not necessarily industrialized. But the dominant GM products are of this kind, at least up to now, and these are the kind of GM food or feed plants that have been assessed under the GTA so far. However, the critique of GM food is not only a matter of conflicting ideals of food production. Underlying these cultural ideals are questions of economy and control in the food industry.

### The Social and Ethical Dimension of Power and Control

Negative attitudes to GM crops among consumers in Norway and Europe (Bugge and Rosenberg 2017; Gaskell et al. 2010; Magnus et al. 2009) are commonly assumed to be related to the perceived health risks of GM food. These negative attitudes are, however, also expressive of a lack of trust in those who develop and control the dominant applications of the technology, namely multinational agrochemical companies. This was clearly expressed both in a survey and in focus groups in a recent Norwegian study (NBAB 2020). Similar attitudes are expressed in Swedish reports (Fisher et al. 2019). One can discuss to what extent this is a well-placed distrust or the result of unsuccessful public relations work and unfair presentations by others. But it is likely that it is also connected to a suspicion regarding the goals of some of the dominant GM producers.

Surveys have found that in EU countries, consumers are concerned about food security, but they also emphasise issues such as the food’s origin and quality (European Commission 2012), in keeping with the ideals of the specialty food movements. It is also likely that these socio-cultural reasons for skepticism to GM food is closely connected to the concern with naturalness in food production, exemplified by cisgenic modification being more acceptable than transgenic in public surveys (Gaskell et al. 2010; Mielby et al. 2013). This indicates that the degree of perceived human intervention in nature is taken to be important by a significant part of the public.

Another reason for public skepticism may be a concern with the adequacy of regulations (Wunderlich and Gatto 2015; Zilberman et al. 2013) and public control. This interpretation is supported by an analysis of relatively early public resistance to agricultural biotechnology. When the GM technology was introduced as a solution to the challenges of a stable and adequate food production, there were several concerns. The central one became issues of human health and environmental safety, as everybody agreed that this was important. One way to handle these concerns was by using a precautionary approach, although this was controversial. The Precautionary Principle has been criticized for being vague, and therefore unsuitable as a regulatory principle, or for being excessive and preventing beneficial technology development (Sunstein 2005), although these charges have been countered by others (Steel 2015). Regardless of the suitability of precaution for managing risk and uncertainties, it is important to notice that safety concerns were not the only arguments for subjecting this technology to precautionary measures. An early driver for precaution was the fear that GM technology was a potent means for transferring power over food production from democratic institutions to “science, technology and the industries that increasingly control them” (Tait 2001, 185). Arguably, this warrants making stronger demands on issues of public concern in addition to reducing risk to health and environment, in the sense that precaution should not only be taken to prevent harm to health and short-term environmental harm. It is perhaps even more important to ensure that food production, an essential factor in human life, is protected from negative social and long-term environmental impacts.

Patents give wider rights and more power over the use of the product than other plant variety protection under the International Union for the Protection of New Varieties of Plants (UPOV) system. At the outset, this is unproblematic: farmers can choose not to use their products if they find the license conditions unacceptable, and the consumers can refrain from buying them, as long as the products are labelled and the food system allows for real alternatives. Canada has no specific regulation of GMOs and there is no legal requirement to label food based on GMOs, whereas the specific labelling requirements varies in other countries. More important, however, is the argument pointed out above: food is not just any product. It is the basis for everyday survival. Patent on GMOs will shift power from public institutions and local farmers to the patent holders, for the time being mainly multinational biotechnology corporations. The extent to which the power actually is shifted, will depend very much on the application. In a recent study by Helliwell and colleagues (2019), the issue of power was also found to be of importance with the potential use of genome-editing in agriculture. In this study representatives from NGOs within environmental, agriculture and farming were invited. The participants found that genome-editing could expand the power held by agriculture biotechnology corporations on industrial agriculture, farmers and, eventually, on consumers.

One way to overcome this power has been suggested by Zoë Robaey (2016), who argues for a shared moral responsibility based on ownership of the GM seeds that includes both the benefits and hazards. However, Robaey found that the Technology/Stewardship agreement that for example is used by Monsanto does transfer rights and duties but is too limited to include transfer of responsibility for both benefits and potential risks. Another approach to retain public control over food production is to require that the benefits of the patent-protected product is also beneficial for the general public—who mainly takes the role of consumers in this context—and is not harmful for future generations. When considering that some patented traits, notably pest resistance and herbicide tolerance, may have significantly reduced value when the patent expires, the usual social utility justification of patents is less convincing (Timmermann 2015). This warrants that risk assessment is supplemented with a compulsory evaluation of non-safety issues, which subsequently would imply that the company applying for commercial release and use of its product must take these into account. The successful history of the publicly controlled food production system in Norwegian agriculture and breeding programs gives a special cultural support for these demands.

## New Opportunities for Trust and Social Acceptance of Genome-Edited Plants

As genome editing is a more accessible technology to a variety of institutions by being less expensive and more efficient than previous GM technologies, one can imagine that the future will bring a number of niche products either patented or freely available by open source. In addition, genome-edited plants are not only developed by transnational companies but also by smaller, locally based companies and research institutions. Also, in these cases, one can argue that public control should be retained by ensuring that these products are sustainable and give societal benefit, following the tradition of Norwegian breeding programs. This will reduce public GM skepticism (NBAB 2020). In addition, inclusion of non-safety issues can increase people’s trust in regulatory processes. The issue of trust is of crucial significance when it comes to consumer and market acceptance, and concerns both consumers possibility to choose the food they want to buy by providing relevant information, as well as the way the food has been produced.

Social acceptance may also be related to the degree of change and novelty introduced by new technologies, as well as the species modified, and whether the organism is to be used for production of food or pharmaceuticals (Frewer et al. 2013). The degree of change and novelty is particularly relevant for genome-edited products that contain no foreign DNA. They will probably be more acceptable, just like cisgenic products are more acceptable than transgenic for a significant part of the general public (Gaskell et al. 2010; Mielby et al. 2013). However, as pointed out by Richard Helliwell and colleagues (2019), consumers may require a right to information enabling informed food choices.

On this background, there are reasons to hold that one should evaluate both GM and genome-edited plants, including those with no foreign DNA inserted, according to relevant non-safety concerns of the kind that include ethical issues (e.g. animal welfare and animal integrity, consumer autonomy and justice), societal distributions of benefits and sustainability, irrespective of its risk regulation status. When a basic concern in the debate is that technology may alter the social and economic conditions for food production and give a shift in power relations, this can be handled by an assessment of societal benefits with adequate conditions. The European opposition to GM products has for example been explained by the lack of direct benefits for consumers by presently commercially available GM crops (Fresco 2013). Likewise, when the concern is with potential long-term effects on the environment due to altered agricultural practices, a short-term risk assessment is not adequate alone. The issue of sustainability must therefore also be addressed. These concerns are clearly thematized in the ethical debate, the first as social justice and the second as matter of responsibility.

Assuming that the reason for precautionary measures is not primarily potential risks to health and environment, but is expressive of a concern with how the GM crops will impact established and valued farming practices and public control over food production, it follows that these non-safety issues should be assessed even if it is decided that genome-edited plants are exempt from GM risk assessment regulation. There is every reason to believe that commercially interesting genome-edited organisms will be eligible for patenting, given that the procedure itself is patentable. The struggle to control the CRISPR patents demonstrates the belief in the commercial potential of the technology. The fundamental reasons for concern with the long term social and sustainability impact pointed out by Tait (2001), is valid also for genome-editing, irrespective of the question whether it should be subjected to GM risk assessment regulation.

## Arguments Against Non-safety Assessments

Given the arguments above, it follows that if one takes a precautionary approach, a broader assessment of non-safety issues is justified (Myhr and Myskja 2011). What are the arguments for *not* taking such an approach? The most important is that these criteria are vague, arbitrary and subjective (Zetterberg and Björnberg 2017). They are political and not scientific terms (such as risk) and cannot become the subjects of scientific, objective assessments. This gives wide room for abuse, for irrelevant, subjective rejections contrary to fairness in treatment. In addition, the basis for the assessments is context dependent, hence it may shift over time and depend on institutional frames, national protection goals and regional conditions, such as the applied agriculture systems and geographical conditions. This is for example reflected in Austria’s GMO-free stance to protect their small-scale and mostly organic agriculture (Seifert 2008).

One example of how non-safety assessments may change over time, can be illustrated by the sustainability assessment of an herbicide-tolerant GM soy plant. The authorities may find that the variety does not reduce herbicide use, does not increase yields per acre, and the long-term effects of the herbicide on farmworkers and affected eco-systems are unknown, warranting a prohibition. However, a similar sustainability assessment on a later stage when more information is available, could show that the variety actually led to significant reduction in tillage, which reduced CO_2_ emissions and soil erosion, and there is registered reduction in herbicide use as farmers gather experience in effective use that also contribute to improved and more safe working conditions. This information would have altered the sustainability assessment significantly, with a probable positive outcome. How can one weigh these very different sustainability factors, and how can one decide the correct timing and span for such assessment?

Another example of the apparent subjectivity and arbitrariness of non-safety assessments is the challenge in handling incommensurable values, which can be exemplified with the use of gene drives to eradicate malaria. How can one objectively, or at least in a socially acceptable way, weigh the societal benefits of saving numerous lives against the uncertainty regarding ecosystem effects and eradication of a species? This is a choice between incommensurable values; they cannot be traded off against each other, making the final decision appear arbitrary and subjective.

However, rejecting the inclusion of non-safety factors based on lack of knowledge or data, or the incommensurability of values presupposes that avoiding assessment with no clear quantifiable scientific answer is adequate. That is only the case if there is no need for the assessments in question in the first place. The Norwegian authorities have decided, with across-the-spectrum political support, that non-safety assessments are warranted. Under these circumstances, the task of the assessors is to strive for the best approximation, even if there are no precise, quantified basis for the decision.

These are normative decisions, and they should be based on the best arguments available. Even if the participants in the debate cannot agree or achieve consensus, one can reach a sound decision, given that there is an inclusive debate where all relevant arguments are heard, and where trusted, competent people make the final decision. Many have pointed out that risk analysis and management are also value based and ‘subjective’, showing that there is no direct way from facts to decision (Stirling 2007; Wynne 1992; Zetterberg and Björnberg 2017). Judgement and deliberation are needed also in safety assessments to achieve a socially robust result. One should not exaggerate the difference between risk assessment and non-safety assessment of GMOs. Both are evaluation regimes that require some degree of judgement, and the predictability of the outcome will increase with the number of assessments made. The fact that an assessment changes over time due to new information is not unique for non-safety assessments. There are numerous pesticides that once were allowed that now are prohibited due to new knowledge or altered standards for safety assessment. We must also accept the weighing of incommensurable values when deciding on regulation of other technologies, e.g. within transport. Traffic regulation is a trade-off between the freedom of movement and the value of human life. This trade-off is handled differently in different jurisdictions.

As illustrated with the Norwegian example, assessment of non-safety factors has been subject to a learning process reflected in increased stringency as well as greater scope and depth of detail in the assessments, providing an increasingly predictable evaluation regime. Examples include the frameworks for assessments (NBAB 2011, 2014, 2018b), and the lists of questions to applicants that provide direction for their contributions to non-safety assessments.

There are three arguments for exempting genome-edited organisms from GM regulation: (a) the analogy to organisms found in nature, (b) the similarity to organisms originating from mutagenesis techniques, and (c) the excessive regulatory burden placed on GMOs that is stifling innovation as well as global trade of agriculture commodities (Jones 2015). The two first arguments are not decisive for exempting these technologies from non-safety assessments. Although the characteristics of the organisms is crucial in *making* the non-safety assessment, they are not independent reasons for inclusion in the assessment. The *decisive* reason for distinguishing between these products and conventional products, is the socio-economic framing of genome-editing.

The claim by researchers and industry that genome-editing is a particularly powerful tool for altering organisms, indicates that there is an intrinsic relation between the arguments for stronger protection of ownership rights and the disruptive potential by use of this technology compared to conventional methods. If this is a particularly powerful technology, something that warrants patenting, it is not similar to conventional products even if the use of the technology may in some instances lead to identical products. This reason makes the third argument highly significant. If one assumes that the promises of genome-editing will hold true, these technologies will give valuable benefits for everybody, including the environment; not only the patent holders and industrial farms. Excessive regulation will slow down the uptake of these improvements, and people will suffer while waiting for them and their benefits to be realized. This is valid for risk assessment, which is a time consuming and costly process. However, the non-safety assessments, as conducted by the NBAB, is neither. As shown above, these assessments are mainly argumentative in character, and are based on available information about the effect on agricultural practices and societal conditions. Working out these arguments is not trivial, but the assessments do not require extensive studies of the kind required for risk assessments.

Non-safety assessment can be based on relevant information and can be performed in parallel with the risk analysis, given the existence of the operalization reports. The main issues will be the distribution of benefits and the long-term effects. An assessment of non-safety factors will evaluate if genome editing delivers on the promises, and provide arguments to the effect that the products are both socially equitable and give a positive contribution to sustainability.

## Non-safety Assessments: A Universal Requirement?

The argument in this article states that non-safety assessments are feasible and justified within the Norwegian context, but are they only valid for this country? Or should assessments of ethics, societal implications and sustainability be implemented in regulation of GMOs and genome-edited organisms everywhere? The arguments and case analyses presented in this article are mainly based on particular cultural and ethical Norwegian traditions, which means that they do not provide sufficient basis for a general world-wide adoption of the assessments. However, several countries do have analogous traditions to those of Norway, and this gives ground for arguing for the implementation of non-safety assessments within these jurisdictions. There is room for tradition based non-safety measures in the EU Directive 2015/412, indicating that the arguments are relevant within the European context.

One should also note that several of the arguments above are of general validity and may justify that the issue is considered also within jurisdictions that have no similar common tradition for protection of food production. This is a disruptive technology with great potential for societal and environmental impact, and food has a special status in human lives regardless of culture. Thus, non-safety concerns regarding GMOs and NPBTs, including genome editing, are not restricted to Norway or Europe. Whether these concerns should be subject to regulations similar to the Norwegian, however, cannot be decided merely by the arguments in this article.

## Conclusion

This article has discussed whether genome-edited organisms can and should be subjected to non-safety assessments in jurisdictions where this kind of assessment is required for GMOs. The question has been discussed in light of the Norwegian experience with evaluating the sustainability, ethical and societal acceptability required in the Norwegian Act using actual cases. It has been argued that the regulatory practice in Norway shows that such assessments of GMOs or genome-edited organisms are feasible without being unreasonably costly or time-consuming. This leaves the question whether such assessments are justified and should be required in jurisdictions where such assessments are required for GMOs.

The main arguments for including non-safety assessments in the regulation of genome-edited products are:

- First, the ownership issue remains the same as with GMOs: this is a patentable technology (Ledford 2017), although it is yet not clear how that right is affected if the resulting organism also could have been produced by non-patentable methods.
- Second, genome-editing technologies have, independent of whether there is or is no addition of foreign material, the potential for altering characteristics with significant impact on sustainability, societal issues and ethics.

Due to the disruptive nature of this technology, these assessments are also morally and politically justified in countries that have comparable values to Norway regarding agriculture and food. The majority of GMOs and genome-edited organisms are made for food production, which is of particular significance for human life, well-being and culture. In countries like Norway, where the publicly controlled food production systems and breeding programs are widely considered to be the basis for trust, the authorities should ensure that this disruptive technology is not only safe, but also sustainable and beneficial for society. In these countries, non-safety assessments are a way to ensure the ethical justifiability and social acceptability of genome editing in food production demanded of the GTA, even if some of these organisms are considered comparable to non-GMOs in terms of risks. In addition, several of the arguments supporting such assessments are of general validity and may justify that the issue is considered also within jurisdictions that have no similar common tradition for protection of food production.
